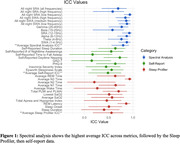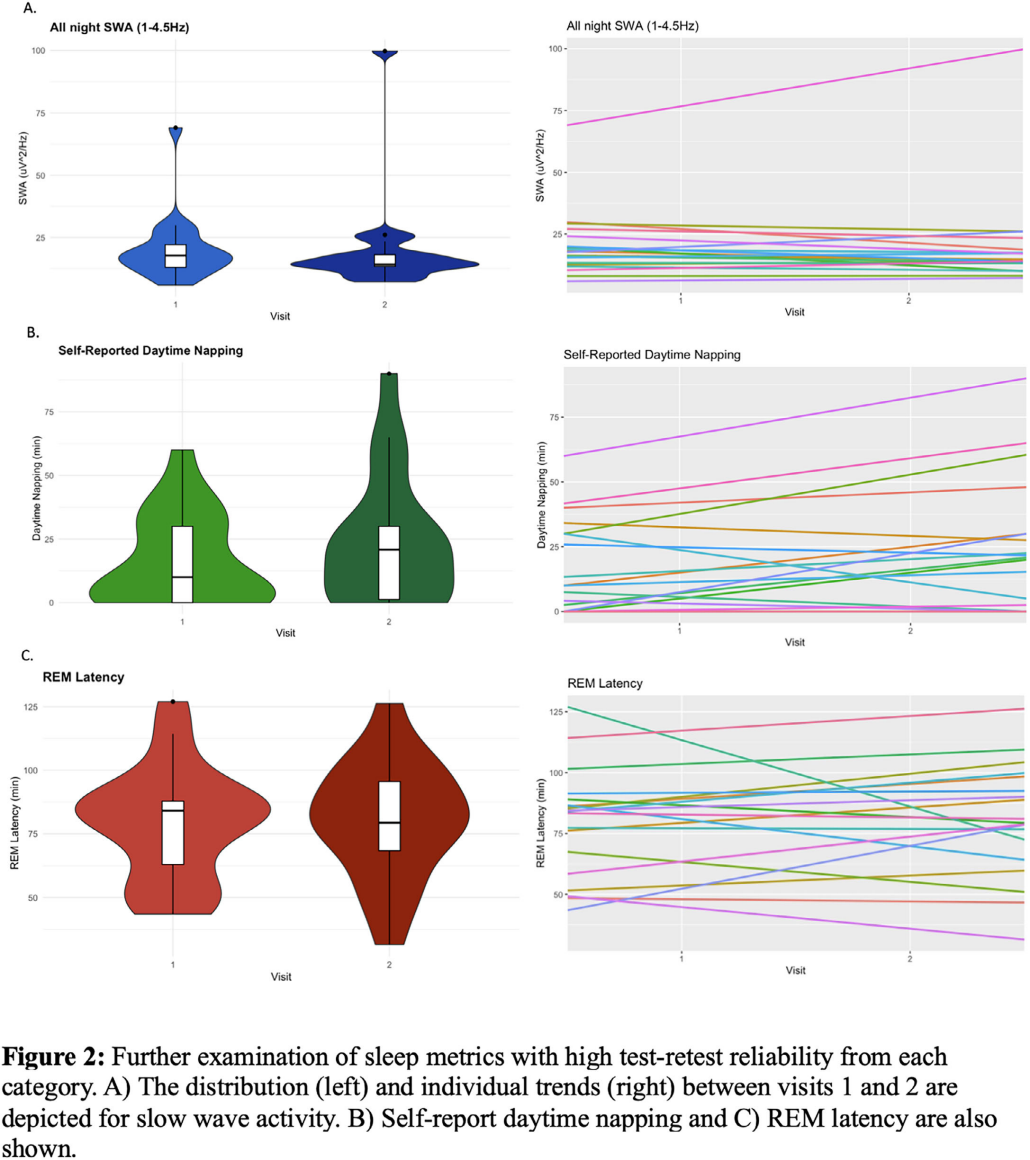# Examination of sleep metrics from spectral analysis, self‐report, and the Sleep Profiler to establish test‐retest reliability in an Alzheimer’s disease cohort

**DOI:** 10.1002/alz.092952

**Published:** 2025-01-09

**Authors:** Taylor J Pedersen, Austin A. McCullough, Cristina D Toedebusch, Ashley Hess, Rachel Richardson, John C. Morris, David M. Holtzman, Brian A. Gordon, Brendan P Lucey

**Affiliations:** ^1^ Washington University School of Medicine, St. Louis, MO USA; ^2^ Washington University in St. Louis, St. Louis, MO USA; ^3^ Washington University School of Medicine, St Louis, MO USA; ^4^ The Charles F. and Joanne Knight Alzheimer Disease Research Center, St. Louis, MO USA; ^5^ Washington University School of Medicine in St Louis, St. Louis, MO USA; ^6^ Washington University in St. Louis School of Medicine, St. Louis, MO USA

## Abstract

**Background:**

It is thought that sleep plays a role in the development and course of Alzheimer’s disease (AD). There are numerous metrics and methods to gather sleep data including measures from self‐reports, actigraphy, or EEG. There has been limited research examining the reliability of these variables over time in healthy individuals as well as the optimal method to gathering such data. We sought out to determine which sleep metrics are the most reliable over a long timescale in a cohort of amyloid negative individuals.

**Method:**

Sleep was assessed over six nights at home using three instruments simultaneously: single‐channel EEG (scEEG) (Sleep Profiler, Advanced Brain Monitoring, Carlsbad, CA), actigraphy (Actiwatch2, Philips Respironics, Andover, MA), and sleep diaries. Participants returned three years later for their second visit using the same sleep assessments. Longitudinal data was analyzed for 19 cognitively normal individuals (CDR = 0) who were amyloid negative by PET. To determine the most reliable method of gathering sleep data, we grouped the metrics into three categories: spectral analysis, self‐reports, and Sleep Profiler data. We then examined the intraclass correlation coefficient (ICC) between visits as a measure of reliability.

**Result:**

Spectral analysis demonstrated the highest test‐retest reliability (mean ICC = 0.798, min. ICC = 0.578, max. ICC = 0.877) followed by the sleep profiler data (mean ICC = 0.558, min. ICC = 0.405, max. = 0.932). Self‐report data exhibited the lowest test‐retest reliability (mean ICC = 0.485, min. ICC = 0.150, max. ICC = 0.729) (Fig. 1). In examining metrics with high ICC values from each category, individual slow wave activity (SWA) data remained highly consistent between visits (ICC = 0.874) (Fig. 2A). REM Latency was the strongest sleep profiler metric and had an ICC value of 0.862 (Fig. 2C). Within self‐report data, the daytime napping questionnaire had the highest test‐retest reliability (ICC = 0.729) (Fig. 2B).

**Conclusion:**

Spectral analysis proved to be the most reliable metric in assessing sleep in amyloid‐negative individuals. Given their lower reliability the Sleep Profiler and self‐report questionnaires are likely not sufficient to evaluate sleep quality alone. Further analysis in amyloid‐positive individuals is being conducted.